# Melatonin abolished proinflammatory factor expression and antagonized osteoarthritis progression in vivo

**DOI:** 10.1038/s41419-022-04656-5

**Published:** 2022-03-07

**Authors:** Shan-Chi Liu, Chun-Hao Tsai, Yu-Han Wang, Chen-Ming Su, Hsi-Chin Wu, Yi-Chin Fong, Shun-Fa Yang, Chih-Hsin Tang

**Affiliations:** 1 Department of Medical Education and Research, China Medical University Beigang Hospital, Yunlin, Taiwan; 2 Department of Orthopedic Surgery, China Medical University Hospital, Taichung, Taiwan; 3 Department of Sports Medicine, College of Health Care, China Medical University, Taichung, Taiwan; 4 Graduate Institute of Biomedical Sciences, China Medical University, Taichung, Taiwan; 5 Department of Urology, China Medical University Hospital, Taichung, Taiwan; 6 School of Medicine, China Medical University, Taichung, Taiwan; 7 Department of Urology, China Medical University Beigang Hospital, Beigang, Yunlin, Taiwan; 8 Department of Orthopaedic Surgery, China Medical University Beigang Hospital, Yunlin, Taiwan; 9 Institute of Medicine, Chung Shan Medical University, Taichung, Taiwan; 10 Department of Medical Research, Chung Shan Medical University Hospital, Taichung, Taiwan; 11 Chinese Medicine Research Center, China Medical University, Taichung, Taiwan; 12 Department of Biotechnology, College of Health Science, Asia University, Taichung, Taiwan

**Keywords:** miRNAs, Chronic inflammation

## Abstract

Progressive structural changes in osteoarthritis (OA) involve synovial inflammation and angiogenesis, as well as activation of the proinflammatory cytokines tumor necrosis factor alpha (TNF-α) and interleukin (IL)-8, and the angiogenic factor vascular endothelial growth factor (VEGF). The endogenous hormone melatonin (*N*-acetyl-5-methoxytryptamine) is involved in antioxidative and anti-inflammatory activities, but how it antagonizes OA progression via its specific receptors is unclear. Here, we demonstrate that the MT_1_ melatonin receptor, but not the MT_2_ receptor, is highly expressed in normal tissue and only minimally in OA tissue. By targeting the MT_1_ receptor, melatonin reversed OA-induced pathology and effectively reduced levels of TNF-α, IL-8, and VEGF expression in OA synovial fibroblasts and synovium from rats with severe OA. Interestingly, we found that the anabolic activities of melatonin involved the MT_1_ receptor, which upregulated microRNA-185a through the PI3K/Akt and ERK signaling pathways in OA synovial fibroblasts. Our investigation confirms the role of the MT_1_ receptor in melatonin-induced anti-catabolic effects in OA disease.

## Introduction

Osteoarthritis (OA) is a common joint disease that involves cartilage degradation, bone remodeling, osteophyte formation, angiogenesis, and synovial inflammation [1]. In the early stages of OA, synovitis is characterized by hyperplasia and inflammation of the synovial membrane [2]. As the disease progresses, OA synovium histology is characterized by the release of increasingly higher amounts of proinflammatory cytokines (IL-1β, TNF-α, and IL-8) from synovial fibroblasts. These cytokines destroy cartilage and stimulate the release of synovial fluids that amplify the inflammatory response [3]. The disease process is stimulated still further in the inflammatory synovium by production of the proangiogenic factor VEGF, which mediates catabolic responses [4, 5], promoting angiogenesis and attracting inflammatory cells (macrophages, B and T cells) to the site of inflammation [6, 7]. No cure exists as yet for OA. Nonsteroidal anti-inflammatory drugs (NSAIDs) are widely used for the management of OA symptoms [8], but deleterious side effects are reported with long-term use [9]. In particular, NSAIDs accelerate the breakdown of OA articular cartilage by inhibiting the proliferation of chondrocytes [10] and suppressing synthesis of cellular matrix components [11], glycosaminoglycans [12], collagen [13], and prostaglandins [14]. Moreover, NSAIDs accelerate radiographic knee and hip OA progression [15]. It is hoped that the development of an alternative substance from natural sources could be protective of cartilage and be safer than currently prescribed pharmacotherapies in the long-term treatment of OA.

The endogenous hormone melatonin (*N*-acetyl-5-methoxytryptamine) may be one such substance. Melatonin exhibits beneficial anti-inflammatory and antioxidant effects in various inflammatory autoimmune diseases [16] and our laboratory has previously reported anti-inflammatory activities of melatonin in rheumatoid arthritis (RA) disease [17]. The involvement of melatonin in key physiological processes in mammals (e.g., the circadian rhythm, oncogenesis, and immune defense activities) is mediated by its binding to high-affinity specific receptors (MT_1_ and MT_2_), enabling melatonin to stimulate signaling pathways and regulate physiological effects [18, 19]. Increasing evidence describes the participation of MT_1_ receptors in neuropsychopharmacological disorders [20], type 2 diabetes [21], glaucoma [22], and cancer progression [23]. One group of researchers has found that RA synovial macrophages express melatonin-specific sites [24], while a Korean group has recorded a statistically significant higher correlation between the melatonin receptor type 1B (*MTNR1B*) polymorphism and rheumatoid factor in RA patients [25]. We have previously documented decreased levels of MT_1_ in RA synovial tissue compared with levels in healthy non-RA clinic samples, and we have described how the MT_1_ receptor is required for melatonin to inhibit TNF-α and IL-1β production [17].

Besides regulating various physiological processes, melatonin is involved in anti-aging and antioxidative activities [26, 27]. Moreover, in experimental diabetic neuropathy, melatonin reduces levels of proinflammatory cytokines in sciatic nerves by downregulating nuclear factor-kappa B (NF-κB) and decreasing inflammatory responses [28]. Moreover, melatonin lowers IL-8 production in human pulmonary fibroblasts [29]. What role the MT_1_ receptor may play in OA has not been explored until now.

In OA, melatonin effectively reduces the effects of IL-1β upon the production of MMP-3 and MMP-13 in chondrocyte cells [30]. Furthermore, melatonin activates the TGF-β signaling pathway to promote cartilage matrix synthesis [31] and attenuates the activity of proinflammatory cytokines (TNF-α and IL-1β) in a rabbit model of OA [29]. It is established that OA synovial fibroblasts (OASFs) perpetuate cartilage degradation by releasing soluble mediators [32, 33]. Therefore, in early-stage OA, synovium‑targeted therapy can alleviate disease-related symptoms and perhaps also prevent structural progression [17, 34]. Up until now, the action of melatonin in OASFs has not been determined.

We describe a significant negative correlation between levels of the MT_1_ receptor and TNF-α, IL-8 and VEGF expression in both animal and clinical samples of OA tissue. Knockdown of the MT_1_ receptor reversed the inhibitory effects of melatonin upon the production of these proinflammatory cytokines. We also found that melatonin lowers the levels of these proinflammatory cytokines by upregulating microRNA (miR)-185a production in OASFs via the ERK and PI3K/Akt pathways. In the rat anterior cruciate ligament transaction (ACLT) OA model, melatonin inhibited the release of TNF-α, IL-8 and VEGF, and protected against bone erosion and cartilage degradation. Our findings suggest that melatonin has anti-catabolic activity in OA.

## Results

### MT_1_ receptor levels were significantly and negatively correlated with proinflammatory factor expression in animal and clinical samples

**Fig. 1:**
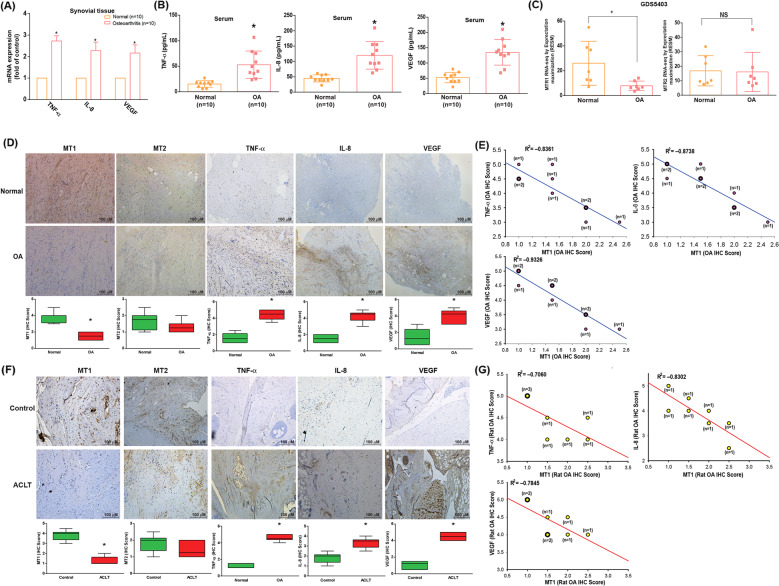
MT_1_ receptor levels were significantly negatively correlated with levels of proinflammatory factor expression in animal and clinical samples. **A**, **B** qPCR and ELISA analyses revealed higher levels of TNF-α, IL-8, and VEGF mRNA and protein expression in human OA synovial tissue (*n* = 10) and OA serum (*n* = 10) than in non-OA samples. **C** Levels of MT_1_ and MT_2_ melatonin receptor expression in 7 paired OA and non-OA tissue samples retrieved from GEO dataset records. **D** The upper panel of IHC staining shows higher levels of MT_1_, MT_2_, TNF-α, IL-8, and VEGF expression in synovial tissue from human OA (*n* = 10) than levels in non-OA (*n* = 10) synovial tissue. The lower panel depicts specimens that were photographed using an optical microscope and scored from 1 to 5 (from weak to strong) for levels of IHC-positive expression. **E** Spearman’s rank correlation coefficient testing identified significant, negative correlations between MT_1_ and TNF-α, IL-8, and VEGF IHC staining scores in synovial tissue retrieved from OA patients (*n* = 10). **F** Histological sections of sham-operated (*n* = 8) and ACLT rat (*n* = 8) synovium stained for MT_1_, MT_2_, TNF-α, IL-8, and VEGF expression (upper panels). IHC scores of MT_1_, MT_2_, TNF-α, IL-8, and VEGF expression (lower panels). **G** Spearman’s rank correlation coefficient testing identified significant, negative correlations between MT_1_ and TNF-α, IL-8, and VEGF scores by IHC staining in synovial tissue from ACLT knee joints (*n* = 8). Error bars indicate means ± S.D. *p < 0.05.

We observed higher mRNA levels of proinflammatory factors TNF-α, IL-8 and VEGF (2.45 ± 0.383, 2.254 ± 0.371, and 2.156 ± 0.227, respectively) in human OA tissue compared with non-OA samples, as well as higher protein levels in human serum compared with non-OA serum (Fig. 1A, B). These findings were supported by immunohistochemical (IHC) staining of human OA and non-OA synovial tissue specimens (*n* = 10 in each group) (Fig. 1D) and the ACLT OA model (Fig. 1F).

No available evidence has as yet discussed the involvement of the MT_1_ receptor in OA inflammation. In our analysis of Gene Expression Omnibus (GEO) data, MT_1_ receptor levels were markedly higher in normal synovial fibroblasts compared with OASFs (Fig. 1C); no such observation was made with the MT_2_ receptor. Similarly, our IHC analyses revealed substantially lower MT_1_ expression in human OA synovium and in synovial tissue from OA (ACLT) rats, whereas MT_2_ levels did not differ between the groups (Fig. 1D, F). When individual IHC staining scores were analyzed by GraphPad Prism 5.0 software, we found negative, significant correlations between MT_1_ receptor expression and levels of TNF-α, IL-8, and VEGF expression in human synovial tissue (Fig. 1E; *n* = 10, Spearman’s *r* = –0.8361, *p* = 0.0014; Spearman’s *r* = –0.8738, *p* = 0.0011; Spearman’s *r* = –0.9326, *p* < 0.0001; respectively) and in OA rat tissue (Fig. 1G; *n* = 8, Spearman’s *r* = –0.7060, *p* = 0.0357; Spearman’s *r* = –0.8302, *p* = 0.0048; Spearman’s *r* = –0.7845, *p* = 0.0321; respectively). We suggest that the marked differences in levels of MT_1_ expression observed between healthy and OA synovium tissue may be a crucial factor underlying inflammation in OA disease, whereas the MT_2_ receptor does not appear to have any such influence.

### The MT_1_ receptor in human OASFs is essential for melatonin-mediated suppression of TNF-α, IL-8, and VEGF expression

**Fig. 2:**
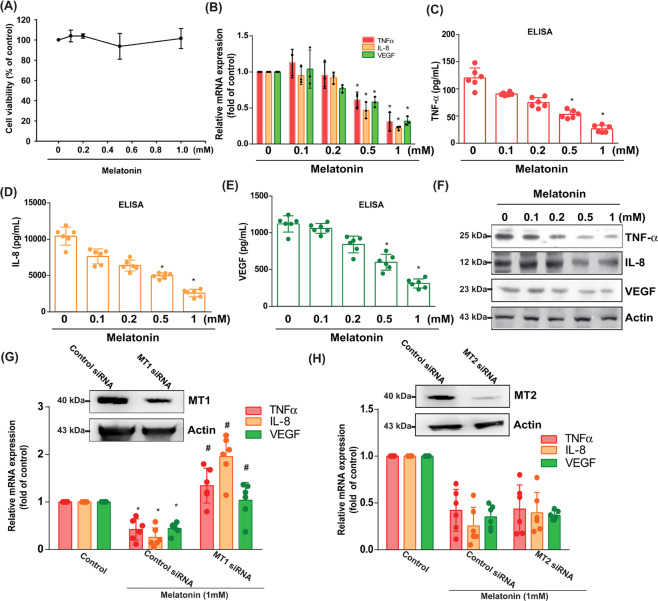
The MT_1_ receptor is essential for the inhibitory effects of melatonin upon proinflammatory factor expression in human OASFs. **A** OASFs were incubated with different concentrations of melatonin for 24 h and the MTT assay measured cell viability. **B**–**F** OASFs were treated with 0–1 mM of melatonin for 24 h. qPCR (*n* = 3), ELISA (*n* = 6) and western blot (*n* = 3) assays quantified TNF-α, IL-8, and VEGF transcription and translation levels. **G**, **H** OASFs were transfected with MT_1_ or MT_2_ siRNAs (*n* = 6) for 24 h, then treated with melatonin 1 mM. TNF-α, IL-8, and VEGF mRNA production was measured by qPCR. Error bars indicate means ± S.D. *p < 0.05.

To further prove that melatonin reduces inflammatory responses, we treated primary OASF cells. We observed that melatonin dose-dependently inhibited TNF-α, IL-8 and VEGF mRNA (0.31 ± 0.075, 0.22 ± 0.015 and 0.32 ± 0.034, respectively) and protein expression, according to qPCR, ELISA and western blot assays (Fig. 2B–F), but did not have any significant effects on IL-1β, IL-6, or IL-17 mRNA levels (Supplementary Fig. S1), or cell viability (Fig. 2A). Furthermore, analysis of clinical OA samples revealed how macrophage infiltration, vascular endothelium proliferation, and higher levels of synovial angiogenesis encourage OA progression. We also discovered that melatonin dose-dependently reduced VEGF mRNA and protein production in OASFs (Fig. 2B–F).

MT_1_ and MT_2_ G-protein-coupled receptors play key roles in physiological effects of melatonin [18, 19]. We used MT_1_ and MT_2_ siRNAs to identify the involvement of MT_1_ and MT_2_ receptors in melatonin-induced inhibition of TNF-α, IL-8 and VEGF expression. We found that knockdown of MT_1_, but not MT_2_, reversed the effects of melatonin (Fig. 2G, H).

### ERK and PI3K/Akt signaling pathways are associated with melatonin-induced inhibition of TNF-α, IL-8, and VEGF production

**Fig. 3:**
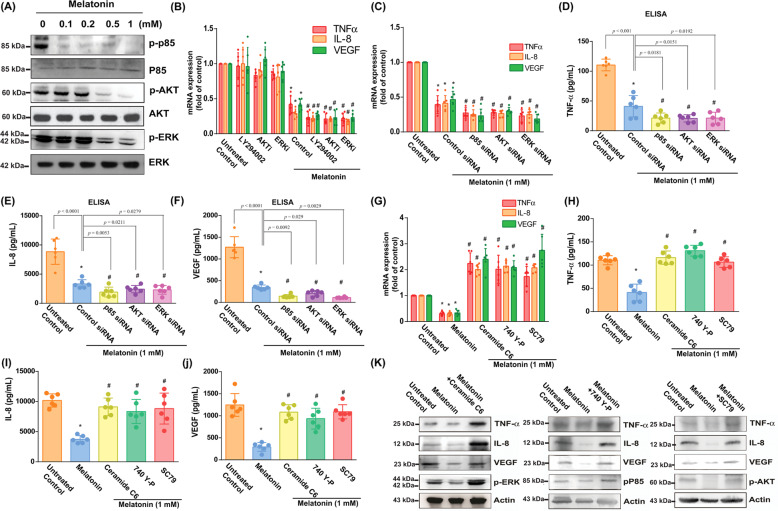
ERK and PI3K/Akt signaling pathways are involved in melatonin-induced downregulation of proinflammatory factors (TNF-α, IL-8, and VEGF). **A** OASFs were incubated with melatonin (0–1 mM) and the Western blot assay (*n* = 3) determined p85, Akt, and ERK phosphorylation. **B** OASFs were treated with pathway inhibitors for 30 min then incubated with 1 mM of melatonin. The qPCR assay (*n* = 6) quantified TNF-α, IL-8, and VEGF expression. **C**–**F** OASFs were transfected with siRNAs then incubated with melatonin (1 mM) overnight. qPCR (*n* = 6) and ELISA (*n* = 6) assays measured TNF-α, IL-8, and VEGF mRNA and protein expression. **G**–**K** OASFs were pretreated with activators of ERK (C6-ceramide) 10 μM, PI3K (740-YP) 10 μM, and Akt (SC-79) 10 μM for 30 min, then incubated with melatonin (1 mM). qPCR (*n* = 6), ELISA (*n* = 6) and western blot (*n* = 3) assays determined the expression of TNF-α, IL-8, and VEGF, respectively. Error bars indicate means ± S.D. *p < 0.05 versus controls; ^#^*p* < 0.05 versus the melatonin-treated group.

Our western blot results show that melatonin dose-dependently reduced levels of p85, Akt, and ERK phosphorylation (Fig. 3A). Pretreatment with PI3K, Akt, and ERK inhibitors (Ly294002, AKTi, and ERKII, respectively) augmented the inhibitory effects of melatonin (Fig. 3B), as did transfection with the respective siRNAs (Fig. 3C–F). Furthermore, incubating the OASFs with these pathway activators reversed these effects of melatonin on TNF-α, IL-8, and VEGF mRNA and protein expression (Fig. 3G–J), by increasing ERK, p85, and Akt phosphorylation (Fig. 3K).

### Melatonin upregulated miR-185a expression and attenuated production of proinflammatory factors

**Fig. 4:**
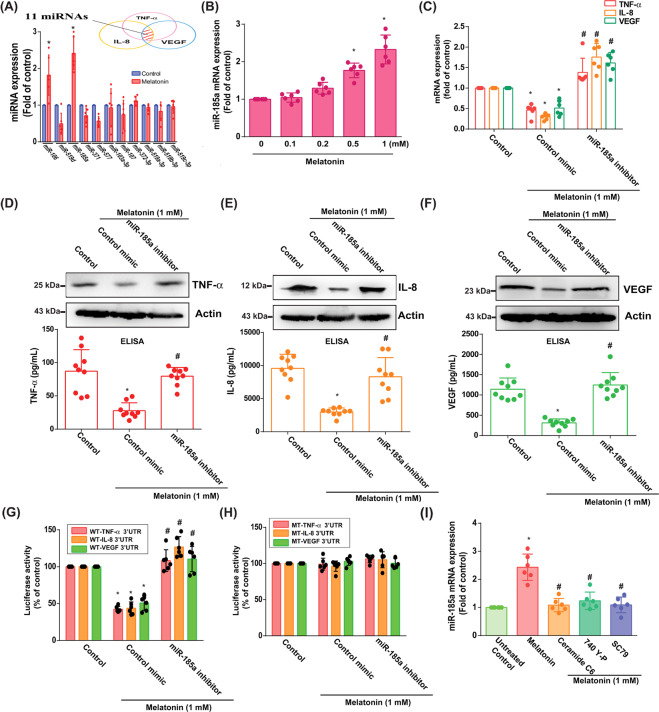
Melatonin upregulated miR-185a expression and attenuated proinflammatory factor production. **A** Using 4 open-source software databases, we predicted that 11 miRs could bind the 3’-UTRs of TNF-α, IL-8, and VEGF. After treating OASFs with melatonin (1 mM) for 24 h, miR levels were measured (*n* = 6). **B** OASFs were treated with different concentrations of melatonin for 24 h, then analyzed by qPCR for miR-185a expression (*n* = 6). **C**–**F** OASFs were transfected with the miR-185a inhibitor for 24 h then incubated with 1 mM of melatonin. qPCR (*n* = 6), western blot (*n* = 3) and ELISA (*n* = 9) assays determined TNF-α, IL-8, and VEGF levels, respectively. **G**, **H** OASFs were transfected with the indicated WT (**G**) or MT (**H**) luciferase plasmids, with or without the miR-185a inhibitor, then stimulated with melatonin (1 mM). Relative luciferase activity (*n* = 6) was determined. **I** OASFs were treated with activators (740-YP, SC-79, or ceramide C6) for 30 min then incubated with 1 mM of melatonin. The qPCR assay (*n* = 6) quantified miR-185a expression. Error bars indicate means ± S.D. *p < 0.05 versus controls; ^#^*p* < 0.05 versus the melatonin-treated group.

To clarify which miRs are involved in OA progression, we examined open-source software databases (TargetScan, miRWalk and RNAhybrid). Eleven candidate miRs were bound to TNF-α, IL-8, and VEGF mRNA 3′-UTR regions, but only miR-185a and miR-106 levels were significantly increased after melatonin 1 mM administration (Fig. 4A). However, treatment of OASFs with a range of melatonin doses (0–1 mM) concentration-dependently enhanced miR-185a expression (1.0, 1.045 ± 0.05, 1.29 ± 0.06, 1.77 ± 0.08, and 2.326 ± 0.15, respectively) (Fig. 4B) but not miR-106 (1.0, 0.89 ± 0.08, 1.12 ± 0.1, 0.72 ± 0.1, and 1.72 ± 0.22, respectively) (Supplementary Fig. S2). To determine whether melatonin inhibits these proinflammatory factors by regulating miR-185a expression, we transfected OASFs with an miR-185a inhibitor. This reversed the effects of melatonin on mRNA expression (Fig. 4C) and protein synthesis of TNF-α, IL-8, and VEGF (Fig. 4D–F).

We used luciferase reporter plasmids containing the wild-type TNF-α, IL-8, and VEGF mRNA-3′-UTRs and mismatches in those target regions predicted for miR-185a (MT-TNF-α, MT-IL-8, and MT-VEGF, respectively), to evaluate the extent to which miR-185a regulates *TNF-α, IL-8*, and *VEGF* gene transcription (Fig. 4G).We found that both the miR-185a mimic and the miR-185a inhibitor reversed melatonin-induced inhibition of luciferase activity in all WT-3′-UTR plasmids, but not in the MT-3′-UTR plasmids (Fig. 4G, H). Incubation with PI3K, Akt, and ERK activators antagonized the effects of melatonin on miR-185a expression (Fig. 4I).

### Melatonin ameliorates synovial inflammation and cartilage degradation in the ACLT model of OA

**Fig. 5:**
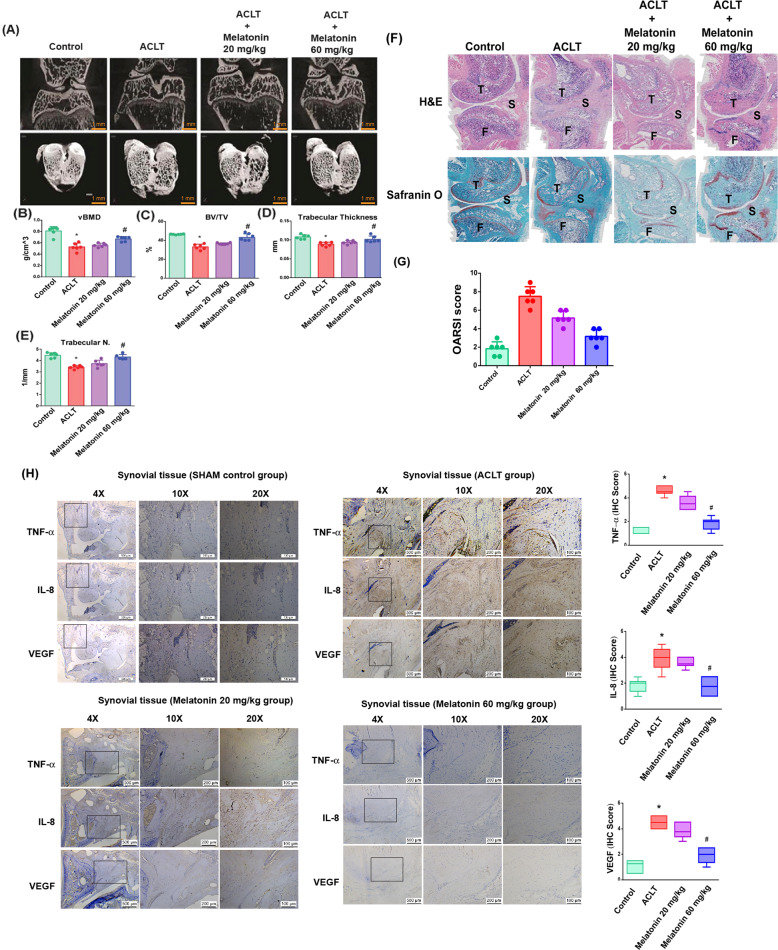
Melatonin ameliorated synovium inflammation and cartilage degradation in the ACLT OA animal model. **A** Photomicrographs of a control knee, ACLT knee, and melatonin-treated knee, with coronal and transverse views from micro-CT imaging (*n* = 6). **B**–**E** Graphic illustrations of vBMD, bone volume, trabecular bone thickness, and trabecular numbers in the different study groups. **F**, **G** Histological sections from knees (*n* = 6) stained with H&E and Safranin-O and associated OARSI scores (S synovium, T tibial, F femoral). **H** IHC staining images from each group showing low- to high-power fields of view for synovial tissue from the same position of joint and quantification of IHC scores. *p < 0.05 versus the control group; ^#^*p* < 0.05 versus the ACLT-only group.

Micro-CT images in coronal and transverse planes from ACLT knees (Fig. 5A) revealed that melatonin treatment (Melatonin 60 mg/kg group) significantly increased bone mineral density (vBMD, Fig. 5B), bone volume (BV/TV, Fig. 5C), trabecular thickness (TT, Fig. 5D) and trabecular number (Trabecular N., Fig. 5E) compared with the ACLT group. Melatonin dose-dependently reduced Osteoarthritis Research Society International (OARSI) scores and the extent of cartilage damage (Fig. 5F, G). Consistent with the in vitro data, IHC staining revealed that melatonin significantly reduced TNF-α, IL-8, and VEGF expression in ACLT-treated knees compared with knees subjected to ACLT alone (Fig. 5G).

## Discussion

OA pathogenesis is multifactorial and remains poorly understood, but it is generally believed that synovial inflammation is pivotal to its pathogenesis [35] and that synovium-targeted therapy can mitigate OA progression and lessen symptomatic disease severity [36, 37]. Angiogenesis and inflammation in the synovium are well recognized as key drivers of OA pathogenesis [32, 33], which is supported by our evidence showing significantly higher levels of proinflammatory cytokine expression (TNF-α and IL-8) and also VEGF, a potent angiogenic factor, in synovial tissues and serum samples from OA patients compared with non-OA clinical samples (Fig. 1).

The various effects induced by melatonin in different cell systems, tissues, organs, humans and rodents, occur via MT_1_ or MT_2_ receptors [38]. The participation of MT_1_ receptors is recognized in neurodegenerative disorders, particularly Alzheimer’s disease [39], although the roles of these receptors are uncertain in OA. Our previous findings of significantly lower levels of MT_1_ expression in human RA synovial tissue compared with healthy, non-RA tissue [17] were also observed in human OA and normal synovial specimens in this study. Our evidence supports an important role for the MT_1_ receptor in melatonin-induced suppression of TNF-α, IL-8, and VEGF expression in human OA synovium. The IHC data in this study verified substantially higher MT_1_ expression in synovial tissue from normal compared with human and rat OA synovium. Moreover, downregulation of MT_1_ expression restored melatonin-induced suppression of TNF-α, IL-8, and VEGF production in OA synovial tissue. It appears that melatonin inhibits TNF-α, IL-8, and VEGF production through the MT_1_ receptor.

Dysregulated miR expression is associated with many human diseases, including OA [40]. Notably, overexpression of miR-140-3p, miR-140-5p and miR-146a in OA cartilage downregulates the expression of important inflammatory mediators, restores homeostatic cellular mechanisms and strongly inhibits inflammation caused by IL-1β and TNF-α [41]. Novel therapeutic approaches (including melatonin) that upregulate the expression of miR-432 and miR-3150a-3p in OA or RA synovial tissue or that downregulate miR-145 expression in chondrocyte cells imply that reducing levels of inflammation is helpful in the management of OA [17, 34, 42]. When we used open-source software miR prediction databases to predict which miRs are related to OA, our investigations revealed that melatonin upregulates miR-185a sequences complementary to the TNF-α, IL-8, and VEGF 3′-UTRs in OASFs. In OASFs, melatonin-induced inhibition of TNF-α, IL-8, and VEGF production was restored by treatment with a miR-185a inhibitor. Our study data emphasize the important role of miR-185a in the inhibitory effects of melatonin on proinflammatory and angiogenic activity in OA.

Melatonin exerts anti-inflammatory activities in several disease states, including OA [43, 44]. In an animal model of diabetic neuropathy, melatonin-induced reductions in pro-inflammatory factor expression led to reduced inflammatory responses, via suppressed NF-κB signaling [28]. In a mouse model of acute gastric ulcer, melatonin effectively downregulated myeloperoxidase activity and TNF-α, IL-1β, and IL-6 cytokine expression [45]. In OA chondrocytes, melatonin effectively suppresses proinflammatory cytokines and IL-1β-induced expression of MMP-3 and MMP-13 [30]. Up until now, the effects of melatonin in OASFs have been undefined. According to our evidence, melatonin significantly lowers TNF-α, IL-8 and VEGF expression in OASFs. This finding is similar to that of previous research showing that melatonin effectively inhibits the production of proinflammatory cytokines during lung inflammation, by downregulating the ERK and PI3K/Akt pathways [29]. Our results also show that melatonin dose-dependently inhibits PI3K, Akt, and ERK phosphorylation. Treating OASFs with PI3K, Akt, and ERK activators reduced the function of melatonin upon miR-185a, TNF-α, IL-8 and VEGF expression. These findings indicate that melatonin enhanced miR-185a expression via PI3K/Akt and ERK signaling.

Our in vitro data were supported by the preclinical investigations showing that melatonin treatment significantly lowered TNF-α, IL-8, and VEGF expression in synovium from ACLT-treated knees. The alleviation of disease activity in the ACLT OA animal model indicates that melatonin is protective of cartilage and bone, but how melatonin affects these parameters in the sham control group needs more experimental evidence. Furthermore, of the many surgically-induced OA models, the ACLT model is the most commonly used in OA research today. The results are highly reproducible and OA disease progresses rapidly. However, a disadvantage of this rapid induction is that it may be too fast to discern early stages in OA development and to detect early effects of drug treatment. Future experiments are called for that include other OA animal models, including naturally occurring disease (*STR/ort* mice; mice exhibiting naturally occurring OA) or age-related OA animal models, for further elucidation of the effects of melatonin in OA progression [46].

A limitation of our study is that we did not examine MT_1_ expression in different Ahlbäck radiographic grades, to ensure that the MT_1_ receptor is negatively correlated with OA progression. Furthermore, to maintain patient confidentiality, no demographic details or any other personal information was recorded for our study participants, which prevented us from comparing demographic details with levels of MT_1_, TNF-α, IL-8, and VEGF expression.

**Fig. 6:**
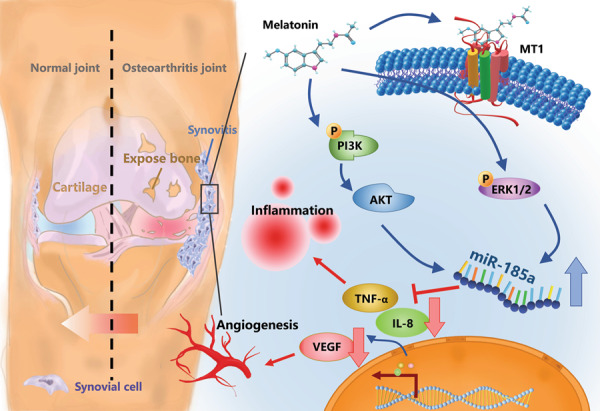
The schematic diagram summarizes the mechanism whereby melatonin inhibits TNF-α, IL-8, and VEGF production. Melatonin attenuates TNF-α, IL-8, and VEGF expression by targeting the MT_1_ receptor and thus inhibits the PI3K/Akt and ERK signaling pathways, leading to upregulation of miR-185a expression in human and rat OA synovium.

Targeting the inflamed synovium slows OA progression and lessens symptom severity [37, 47–49]. Our study is the first to demonstrate that targeting the MT_1_ receptor enables melatonin to effectively lower TNF-α, IL-8, and VEGF expression in OASFs and in ACLT-induced OA. Melatonin upregulates miR-185a expression by inhibiting PI3K/Akt and ERK signaling in OASFs (Fig. 6). This study elucidates the mechanisms used by melatonin to inhibit the release of proinflammatory factors and ameliorate bone loss and cartilage degradation in OA, all of which strongly suggests that this endogenous molecule could usefully treat OA.

## Materials and methods

### Clinical samples

Serum and synovial tissue samples were obtained from 10 patients with radiographically-detected OA of the knee classified by Ahlbäck criteria as stage IV OA [50] during total knee replacement surgery, and from 10 patients undergoing arthroscopy for trauma/joint derangement, who served as normal controls. The study protocol was approved by the Institutional Review Board (IRB) of China Medical University Hospital. All study participants signed written informed consent forms prior to study participation.

### Primary OASF cultures

Synovial tissue used in the primary OASF cultures were obtained from the same donors referred to in the previous section. After undergoing several washes with PBS, the synovial tissue was digested for 5 h at 37 °C by collagenase solution (6 mg/mL) (Sigma-Aldrich, St. Louis, MO, USA) to dissociate the cells from the extracellular matrix. After 6 h, the cell pellet was washed with PBS and centrifuged for 10 min prior to culturing in DMEM medium with 10% of FBS (Invitrogen, Carlsbad, CA, USA). Experiments were performed using cells from passages 3–6.

### Analysis of the Gene Expression Omnibus (GEO) database

Synovial tissue samples from 7 normal healthy donors and from 7 patients with OA were retrieved from the GEO database (accession code: GDS5403).

### Cell viability assay

Melatonin was dissolved in EtOH solution and melatonin cytotoxicity was determined by the 3-(4,5-dimethylthiazol-2-yl)-2,5-diphenyltetrazolium bromide (MTT) assay (50 mg/mL) (Sigma-Aldrich, St. Louis, MO, USA). OASFs were seeded at a density of 5 × 10^3^ cells/well in 96-well plates and treated with melatonin (0–1 mM) for 24 h, then added to the MTT solution. After 2 h, dimethyl sulfoxide (DMSO) was added to the solution and absorbance was determined at 550 nm using a microplate reader (Bio-Tek, Winooski, VT, USA).

### Quantitative real-time PCR (qPCR) of mRNA and miR

mRNA from OASFs and synovial tissue was isolated from OA patients and healthy donors using TRIzol™ Reagent. Complementary DNA (cDNA) was synthesized by the MMLV reverse transcription system (Invitrogen, Carlsbad, CA, USA) and mixed with Fast SYBR^®^ Green Mix. Gene expression was assayed using the StepOnePlus^™^ Real-Time PCR System. The MMLV reverse transcription system (Invitrogen, Carlsbad, CA, USA) synthesized cDNA from total RNA and the FAST SYBR^®^ RT-PCR kit was used to detect miRs. Relative mRNA expression was calculated using the 2^–△△Ct^ method, with GAPDH as the internal reference. The primers used in the qPCR assays are listed in Supplementary Table 1.

### Western blot analysis

OASFs were washed in PBS and then the proteins were dissolved with RIPA buffer containing protease inhibitors. Protein concentrations were determined using a BCA Protein Assay kit. Proteins (30 µg/lane) were separated by SDS-PAGE gels and then transferred to polyvinyldifluoride (PVDF) membranes (Millipore). The membranes were blocked with Tris-buffered saline (TBS) containing 5% nonfat milk and incubated in specific primary antibodies: anti-p85, Akt, phospho-ERK, and ERK antibodies (Santa Cruz biotechnology, CA, USA), anti-MT_1_, anti-VEGF, and anti-IL8 antibodies (Abcam, Cambridge, MA, USA), as well as anti-TNF-α antibody (Abclonal, MA, USA), then incubated with HRP-conjugated secondary antibodies at room temperature. Proteins were detected in the blots using enhanced chemiluminescence (ECL) reagents (GE Healthcare Life Sciences, UK). Detailed information about the antibodies used in Western blot assays are listed in Supplementary Table 2.

### Enzyme-linked immunosorbent assay (ELISA)

Different concentrations of melatonin (Sigma-Aldrich, MO, USA) and specific inhibitors of Akt (AKTi) (Sigma-Aldrich, MO, USA) 10 μM, ERK (ERK II) (Santa Cruz Biotechnology, CA, USA) 10 μM, and PI3K (Ly294002) (Enzo Biochem, Inc., NY, USA) 10 μM, or activators of ERK (C6-ceramide) 10 μM, PI3K (740-YP) 10 μM, and Akt (SC-79) 10 μM (Santa Cruz Biotechnology, CA, USA) were added to OASFs (during passages 3–6) for 30 min, or the OASFs were transfected with p85, Akt, and ERK siRNAs (Dharmacon, Lafayette, CO, USA) for 24 h before melatonin treatment. After 24 h, conditioned medium was collected and quantified using commercially available proinflammatory factor-specific (TNF-α, IL-8, and VEGF) ELISA kits (R&D, MN, USA), according to the manufacturer’s instructions. The plates were read at 450 nm. Calculations were performed according to the standard curve for the determination of sample concentrations.

### Transient transfection

OASFs were cultured in 6-well plates and transfected with the miR-185-5p inhibitor using Lipofectamine™ 2000. ON-TARGETplus siRNAs targeting p85 (L-003020-00-0005), ERK (L-00355500) and Akt (L-003000-00-0005) were purchased from Dharmacon Research (Lafayette, CO, USA). siRNA (100 nM) was transiently transfected with DharmaFECT1 transfection reagent, according to the manufacturer’s instructions.

### Plasmid construction and luciferase assays

TNF-α, IL-8, and VEGF 3′-UTR wild-type (WT) and mutant (MT) binding sites of miR-185a DNA inserts were subcloned into the pmirGLO luciferase reporter vector (Promega, Madison, WI, USA). The primers used to construct the plasmids are listed in Supplementary Table 1. The mutant VEGF 3′UTR region (MT-VEGF-3′-UTR) was obtained from Invitrogen (CA, USA). Finally, luciferase activity was determined using a Dual-Luciferase Reporter assay system (Promega, Madison, WI, USA) [51, 52].

### Anterior cruciate ligament transaction (ACLT)

Male Sprague Dawley rats (5 months old, 160 ± 20 g) were purchased from Taipei’s National Laboratory Animal Centre (Taiwan). We followed an established protocol for our ACLT rat model to induce OA. The ACL fibers were transected with a scalpel and the entire medial meniscus was excised via the medial parapatellar mini-arthrotomy approach. After surgery (day 0), the ACLT rats were administered intraperitoneal (IP) injections of saline 100 μL (*n* = 8), melatonin 20 mg/kg (*n* = 8), or melatonin 60 mg/kg (*n* = 8) once daily for 6 weeks. Sham-operated rats (controls, *n* = 8) were untreated. Rats could move freely until necropsy at 10 weeks post-surgery.

### Micro-CT analysis

After removing the skin and muscle tissue, the intact knee joint (*n* = 6 each group) was fixed in 3.7% formaldehyde at room temperature. The joint was scanned using the Bruker SkyScan 2211 nano-computed tomography (CT) system (Bruker MicroCT, Kontich, Belgium) at the resolution of 8.5 µm within saline. Micro-CT was performed using cameras that scanned over 180 degrees of rotation, a voltage of 90 kVp, a current of 450 µA (8-watt output) and a 0.5 mm aluminum (Al) filter to prevent beam hardening artifacts. Image reconstruction was performed using InstaRecon software (Bruker MicroCT, Kontich, Belgium), which also performed the ring artifact and beam-hardening corrections. The average grey level intensity of the reconstructed image was measured in both scans and a linear calibration was derived between the grey level intensity and bone mineral density (BMD). In brief, reconstructed cross-sections were re-orientated and 59 slices (0.5 mm) were selected and we drew manual regions of interest (ROIs) of an irregular anatomical contour in the subchondral trabecular bone region for the medial tibial plateau. Thresholding, region-of-interest selection, bone morphometric analysis and BMD, bone volume over total volume (BV/TV), trabecular thickness (TT), and trabecular number (Trabecular N.) analyses were performed using CTAn software (Version 1.20.8, Bruker MicroCT, Kontich, Belgium).

### Histological and immunohistochemical analyses

The histopathological changes were measured by hematoxylin and eosin (H&E) and Safranin-O staining under light microscopy. Cartilage damage was scored separately in a blinded way using a detailed semiquantitative version of the OARSI scoring system, adapted for sagittal sections, to measure structural cartilage changes in the central weight-bearing area of the medial tibial plateau in all samples [34]. In this system, the grade of damage from 0 to 6 is defined as the depth of progression of OA into the cartilage and the stage of damage is defined as the horizontal extent of cartilage involvement from 0 to 4. The final score is the combined value of grade and stage (score range 0–24). This scoring was performed by two independent assessors to minimize observer bias [34, 53].

Human synovial tissue (*n* = 10) and rat cartilage tissue (*n* = 8) sections were incubated for 24 h with MT_1_, MT_2_, VEGF, IL-8, and TNF-α antibodies, then their levels were quantified by IHC analysis. IHC staining was scored from 1 to 5 (from weak to strong) for positive expression by two independent observers who were blinded to the treatment groups. Detailed information about the antibodies used in IHC analyses are listed in Supplementary Table 2.

### Statistical analysis

All statistical analyses were carried out using GraphPad Prism 5.0 (GraphPad Software) and all values are expressed as the mean ± standard deviation (S.D.). Samples were analyzed using the Student’s *t*-test (Fig. 1) or one-way analysis of variance (ANOVA) to compare differences between groups for in vitro analyses and by one-way ANOVA followed by Bonferroni testing for in vivo analyses. The statistical difference was considered to be significant if the *P*-value was <0.05.

## Supplementary information

Supplementary Table 1. Primer sequences for qPCR and plasmid construct

Supplementary Table 2. Antibody for Western blot and immunohistochemistry

Supplementary figure S1

Supplementary figure S2

Supplementary figure S3

Supplementary figure S4

checklist

Supplemental Figure legends

Related Manuscript File

## Data Availability

The data generated and analyzed will be made from the corresponding author on reasonable request. Full, uncropped western blot images are now provided in the Supplementary files (Fig. S3 and 4).
