# Supplementary material for: Melatonin abolished proinflammatory factor expression and antagonized osteoarthritis progression in vivo
Source: Cell Death Dis. 2022 Mar 7;13(3):215. doi: 10.1038/s41419-022-04656-5 (PMC8901806; doi:10.1038/s41419-022-04656-5)
Supplement: Supplementary file 1 — Supplementary Table 1. Primer sequences for qPCR and plasmid construct [file 41419_2022_4656_MOESM1_ESM.doc]

**Supplementary Files**

| **Supplementary Table 1. Primer sequences for qPCR and plasmid construct** | | |
| --- | --- | --- |
| **Gene names** | sense(5’-3’) | Anti-sense (5’-3’) |
| TNF-α | CCTCTCTCTAATCAGCCCTCTG | GAGGACCTGGGAGTAGATGAG |
| IL-8 | CTGCGCCAACACAGAAATTATTGTA | TTCACTGGCATCTTCACTGATTCTT |
| VEGF | GCAGAATCATCACGAAGTGG | GCATGGTGATGTTGGACTCC |
| GAPDH | ACCACAGTCCATGCCATCAC | TCCACCACCCTGTTGCTGTA |
|  |  |  |
| **Construct names** |  |  |
| WT- TNF-α 3’-UTR | CGGCTAGCTGCTGCAGGACTTGAGAAGA | GGCTCGAGGGCTACATGGGAACAGCCTA |
| MT- TNF-α 3’-UTR | GGACCTTAGGCCTTCCTCGAGCCAGATGTTTCCAGACTT | AAGTCTGGAAACATCTGGCTCGAGGAAGGCCTAAGGTCC |
| WT- IL-8 3’-UTR | CGGCTAGCGGGTACCCAGTTAAATTTTCATTTC | GGCTCGAGTTTGGAGAGCACATAAAAACATC |
| MT- IL-8 3’-UTR | CTTAAGATGTTTTTATGTGCTAGACAAATTTTTTTTACTGTTTC | GAAACAGTAAAAAAAATTTGTCTAGCACATAAAAACATCTTAAG |
| WT- VEGF 3’-UTR | CGGCTAGCTGAGATGTATCTTTTGCTCTCTCTTG | GGCTCGAGGTACTACGGAATATCTCGAAAAACTG |
|  |  |  |
