# Supplementary material for: Melatonin abolished proinflammatory factor expression and antagonized osteoarthritis progression in vivo
Source: Cell Death Dis. 2022 Mar 7;13(3):215. doi: 10.1038/s41419-022-04656-5 (PMC8901806; doi:10.1038/s41419-022-04656-5)
Supplement: Supplementary file 2 — Supplementary Table 2. Antibody for Western blot and immunohistochemistry [file 41419_2022_4656_MOESM2_ESM.doc]

**Supplementary Files**

| **Supplementary Table 2. Antibody for Western blot and immunohistochemistry** | | |
| --- | --- | --- |
| Primary Ab names | catalogue | Company |
| p85 | SC-1637 | Santa Cruz biotechnology, CA, USA |
| P-p85 | 4228S | Cell Signaling Technology, MA, USA |
| Akt | SC-5298 | Santa Cruz biotechnology, CA, USA |
| P-Akt | 4060S | Cell Signaling Technology, MA, USA |
| ERK | SC-1647 | Santa Cruz biotechnology, CA, USA |
| P-ERK | SC-7383 | Santa Cruz biotechnology, CA, USA |
| MT_1_ | ab203038 | Abcam, Cambridge, MA, USA |
| MT_2_ | orb331264 | Biorbyt, Cambridge, MA, USA |
| TNF-α | A11534 | Abclonal, MA, USA |
| VEGF | A17877 | Abclonal, MA, USA |
| IL-8 | ab18672 | Abcam, Cambridge, MA, USA |
