# Supplementary figures and images for: Melatonin abolished proinflammatory factor expression and antagonized osteoarthritis progression in vivo
Source: Cell Death Dis. 2022 Mar 7;13(3):215. doi: 10.1038/s41419-022-04656-5 (PMC8901806; doi:10.1038/s41419-022-04656-5)

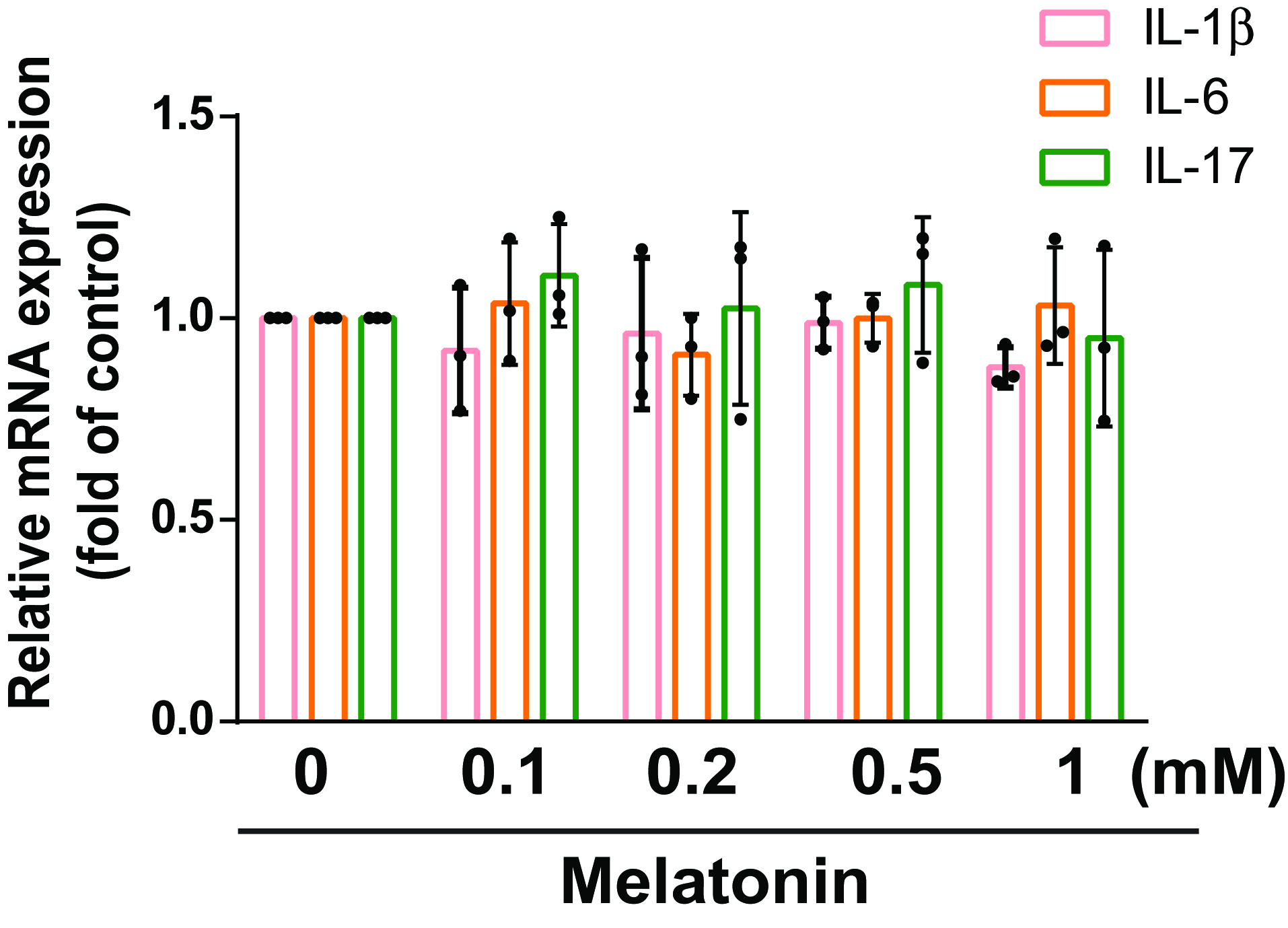

Supplement: Supplementary file 3 — Supplementary figure S1 [file 41419_2022_4656_MOESM3_ESM.tif]

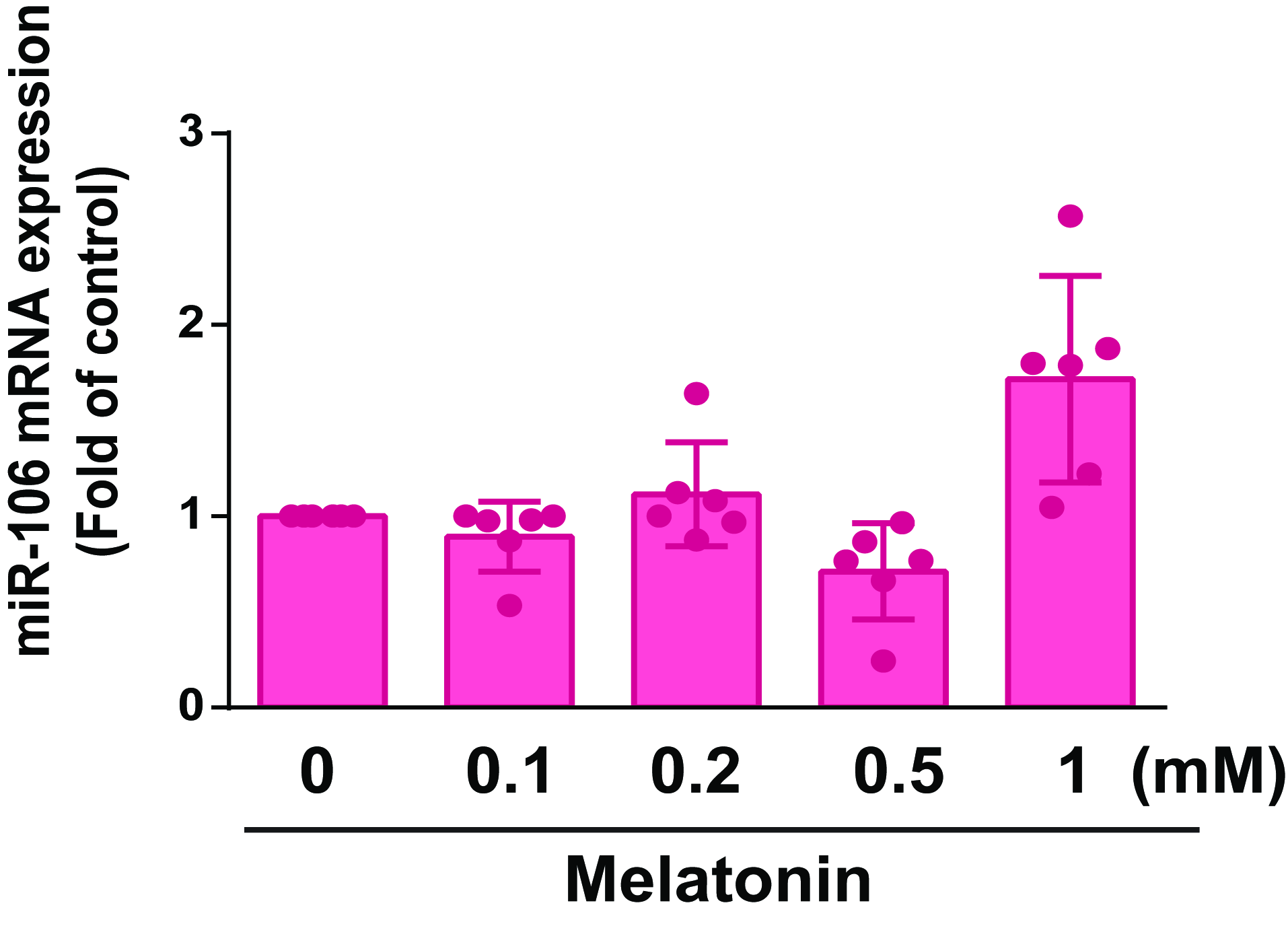

Supplement: Supplementary file 4 — Supplementary figure S2 [file 41419_2022_4656_MOESM4_ESM.tif]

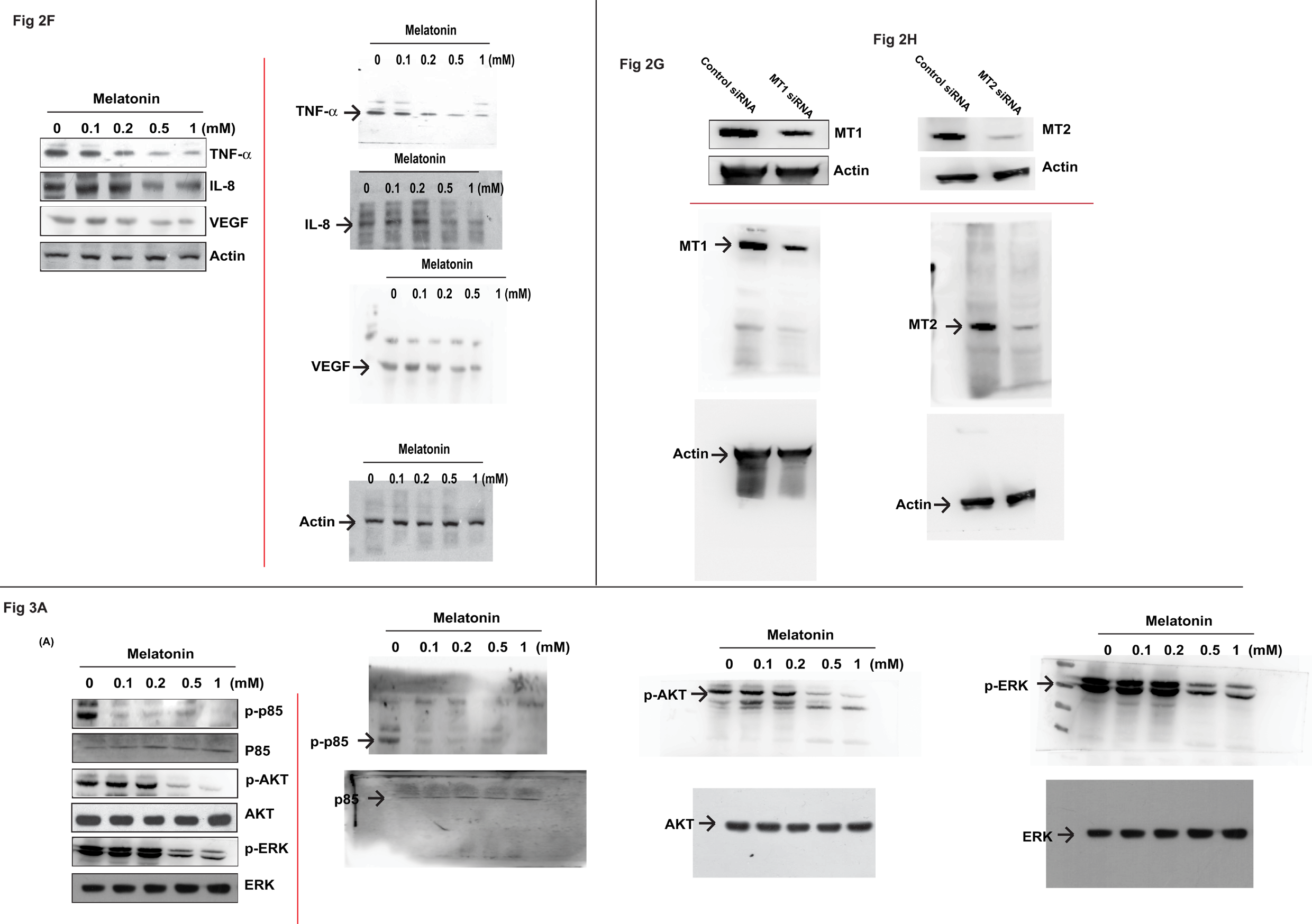

Supplement: Supplementary file 5 — Supplementary figure S3 [file 41419_2022_4656_MOESM5_ESM.tif]

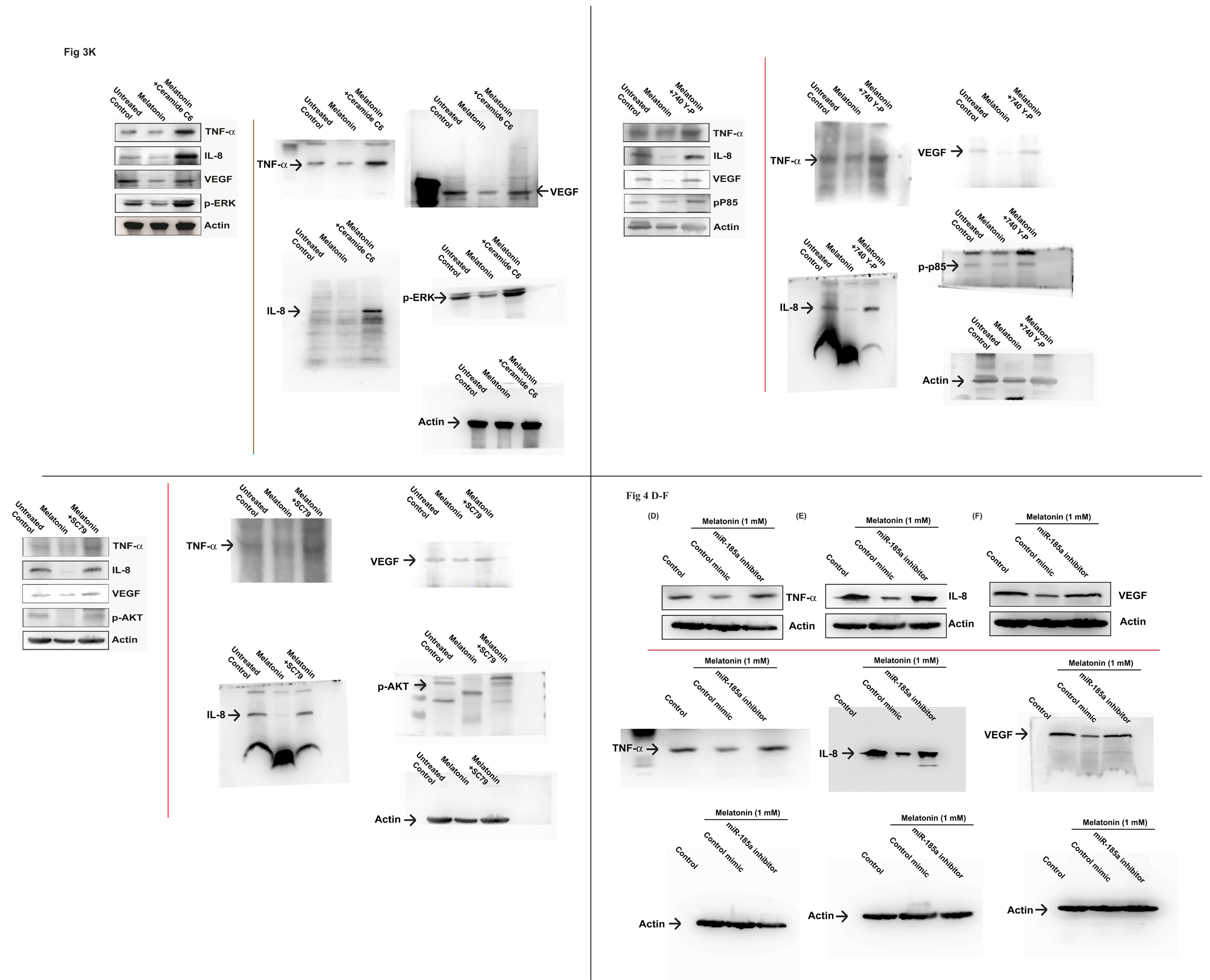

Supplement: Supplementary file 6 — Supplementary figure S4 [file 41419_2022_4656_MOESM6_ESM.tif]
