# Supplementary material for: Melatonin abolished proinflammatory factor expression and antagonized osteoarthritis progression in vivo
Source: Cell Death Dis. 2022 Mar 7;13(3):215. doi: 10.1038/s41419-022-04656-5 (PMC8901806; doi:10.1038/s41419-022-04656-5)
Supplement: Supplementary file 8 — Supplemental Figure legends [file 41419_2022_4656_MOESM8_ESM.docx]

**Supplemental Figure legends**

**Supplementary Table 1. Primer sequences for qPCR and plasmid construct**

**Supplementary Table 2. Antibody for Western blot and immunohistochemistry**

**Supplementary Figure S1. Melatonin did not have any significant effects on IL-1β, IL-6, or IL-17 mRNA levels in OASFs.** Treated OASFs with 0-1 mM of melatonin for 24 h. qPCR assays (n=3) quantified IL-1β, Il-6, and IL-17 transcription levels.

**Supplementary Figure S2. Melatonin did not concentration-dependently enhanced miR-106 expression in OASFs.** OASFs were treated with different concentrations of melatonin (0-1 mM) for 24 h, then analyzed by qPCR for miR-106 expression (n=6).

**Supplementary Figure S3. Uncropped blots for Western blot.** The Fig 2F, 2G, 2H, and 3A uncropped blots.

**Supplementary Figure S4. Uncropped blots for Western blot.** The Fig 3K and 4D-F uncropped blots.
