# Supplementary material for: Melatonin abolished proinflammatory factor expression and antagonized osteoarthritis progression in vivo
Source: Cell Death Dis. 2022 Mar 7;13(3):215. doi: 10.1038/s41419-022-04656-5 (PMC8901806; doi:10.1038/s41419-022-04656-5)
Supplement: Supplementary file 9 — Related Manuscript File [file 41419_2022_4656_MOESM9_ESM.pdf]

DECLARATION OF CONTRIBUTIONS TO ARTICLE

ADMC

Manuscript Number: CDDIS-21-2300

Journal Name: *Cell Death & Disease* (the 'Journal')

Proposed Title of the Contribution:

Melatonin abolished catabolic factor expression and antagonized osteoarthritis progression in vivo (the 'Contribution)

Author(s):

Shan-Chi Liu, Chun-Hao Tsai, Yu-Han Wang, Chen-Ming Su, Hsi-Chin Wu, Yi-Chin Fong, Shun-Fa Yang and Chih-Hsin Tang (the 'Authors')

For all *CDDis* articles, each person named as an author in the published version must be able to show he or she has contributed substantially to the article.

Authorship credit should be based on 1) substantial contributions to conception and design, acquisition of data, or analysis and interpretation of data; 2) drafting the article or revising it critically for important intellectual content; and 3) final approval of the version to be published. Authors should meet conditions 1, 2 and 3.

Any person who cannot be shown to have made a substantial contribution to the article cannot be listed as an author in the final version. The name of any person who is deemed to have made a minor contribution can, however, appear in the Acknowledgments section of the article.

Please complete the table below to indicate the contributions of all named authors to the manuscript.

Author Full Name:	Specification of Contribution to the Manuscript:
Shan-Chi Liu	Results/experiments, Writing
Chun-Hao Tsai	Resources
Yu-Han Wang	Data curation
Chen-Ming Su	Resources
Hsi-Chin Wu	Supervision
Yi-Chin Fong	Resources
Shun-Fa Yang	Supervision
Chih-Hsin Tang	Writing – Review.

Please complete the table below to indicate the contributions of all named authors to the figures.

Figure 1:

Shan-Chi Liu, Chun-Hao Tsai, Yu-Han Wang, Chen-Ming Su, Hsi-Chin Wu, Yi-Chin Fong, Shun-Fa Yang and Chih-Hsin Tang

Figure 2:

Shan-Chi Liu, Yu-Han Wang, Chen-Ming Su, Hsi-Chin Wu, Shun-Fa Yang and Chih-Hsin Tang

Figure 3:

Shan-Chi Liu, Yu-Han Wang, Chen-Ming Su, Hsi-Chin Wu, Shun-Fa Yang and Chih-Hsin Tang

Figure 4:

Shan-Chi Liu, Yu-Han Wang, Chen-Ming Su, Hsi-Chin Wu, Shun-Fa Yang and Chih-Hsin Tang

Figure 5:

Shan-Chi Liu, Yu-Han Wang, Chen-Ming Su, Hsi-Chin Wu, Shun-Fa Yang and Chih-Hsin Tang

Figure 6:

Shan-Chi Liu, Chun-Hao Tsai, Yu-Han Wang, Chen-Ming Su, Hsi-Chin Wu, Yi-Chin Fong, Shun-Fa Yang and Chih-Hsin Tang

Signed for and on behalf of the Author(s):	Print Name:	Date：
Chih-Hsin Tang	Chin-Hsin Tang	2021-09-09
